# Wavelength-Dependent Shaping of Azopolymer Micropillars for Three-Dimensional Structure Control

**DOI:** 10.1021/acsami.3c09264

**Published:** 2023-08-30

**Authors:** I Komang Januariyasa, Fabio Borbone, Marcella Salvatore, Stefano L. Oscurato

**Affiliations:** † Department of Physics “Ettore Pancini”, Università degli Studi di Napoli “Federico II”, Via Cintia 21, 80126 Naples, Italy; ‡ Department of Chemical Sciences, Università degli Studi di Napoli “Federico II”, Via Cintia 21, 80126 Naples, Italy; § Centro Servizi Metrologici e tecnologici Avanzati (CeSMA), Università degli Studi di Napoli “Federico II”, Corso Nicolangelo Protopisani, 80146 Naples, Italy

**Keywords:** 3D microstructures, azopolymers, optical reconfiguration, wettability, light-induced reshaping

## Abstract

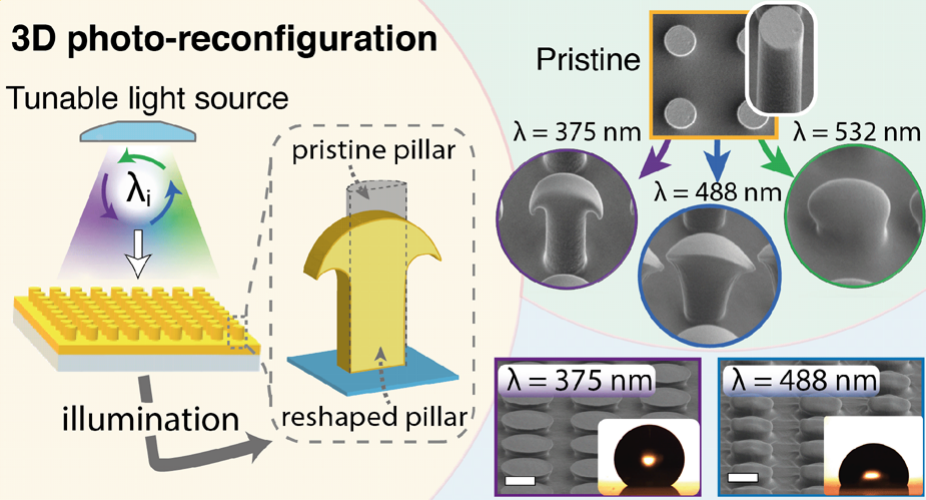

Surfaces endowed with three-dimensional (3D) mesostructures, showing features in the nanometer to micrometer range, are critical for applications in several fields of science and technology. Finding a fabrication method that is simultaneously inexpensive, simple, fast, versatile, highly scalable, and capable of producing complex 3D shapes is still a challenge. Herein, we characterize the photoreconfiguration of a micropillar array of an azobenzene-containing polymer at different light wavelengths and demonstrate the tailoring of the surface geometry and its related functionality only using light. By changing the irradiated light wavelength and its polarization, we demonstrate the fabrication of various complex isotropic and anisotropic 3D mesostructures from a single original pristine geometry. Quantitative morphological analyses revealed an interplay between the decay rate of absorbed light intensity, micropillar volume preservation, and the cohesive forces between the azopolymer chains as the origin of distinctive wavelength-dependent 3D structural remorphing. Finally, we show the potentialities of this method in surface engineering by photoreshaping a single original micropillar surface into two sets of different mesostructured surfaces exhibiting tunable hydrophobicity in a wide water contact angle range. Our study opens up a new paradigm for fabricating functional 3D mesostructures in a simple, low-cost, fast, and scalable manner.

## Introduction

Three-dimensional (3D) surface architectures at the mesoscale enable fundamentally new properties and functionalities for materials that do not generally exist for their planar forms, let alone their bulky counterparts.^1,2^ The influence of the reduced size and the complex surface geometry on optical, mechanical, thermal, acoustic, and electrical properties,^1,3^ has led to the application of engineered 3D mesoscopic structures in a wide variety of fields, including in photonic devices,^4,5^ surface wettability,^6−9^ dry adhesive technologies,^10^ biomedical devices,^11,12^ actuators and sensors,^13^ wearable devices,^14^ and energy storage.^15^

In parallel with providing an ever-increasing number of applications, extensive research efforts have been directed toward developing powerful 3D mesostructure fabrication approaches.^1,3,16,17^ State-of-the-art methods, including extreme ultraviolet lithography, focused ion beam, and electron beam lithography, are capable of producing accurate and complex lateral features with resolutions higher than 10 nm.^18^ Such techniques require multistep serial processes^1,3,19^ that involve chemical or physical etching of a resist,^20^ high costs, and specialized facilities to transfer 2D patterns into simple 3D geometries,^16,21^ which pose limits to production upscaling. In addition, they can raise environmental concerns due to the large amounts of hazardous waste and high-energy consumption.^22,23^ Moreover, the 3D lithographic capabilities of these methods remain limited to simple 3D design.

Fabricating 3D architectures of high and arbitrary complexity requires advanced 3D lithographic technologies, such as multiphoton polymerization lithography, which are versatile but still limited by low throughput and the need for expensive equipment.^24,25^ As a result, it remains challenging to find a process that is both suitable for high-volume production and capable of producing tunable complex 3D features.

Azobenzene-containing materials can provide new strategies for creating controlled 3D mesostructures on a surface based on light-driven mass migration that results from the cyclic photoisomerization of azobenzene chromophores upon exposure to UV–visible light. Typical material systems involve the inclusion of azobenzene molecules in a polymer matrix (azopolymers), although many studies have shown that different material design strategies can be used to tune the efficiency of mass transport.^26,27^ The resulting mesostructure geometry can be controlled by the distribution of the light intensity, the local direction of the electric field (light polarization), and the shape of the irradiated wavefront,^28,29^ opening up the unprecedented possibilities of a fully vectorial photolithography. In addition, a structure inscribed on an azomaterial surface can be erased by either unpolarized light irradiation or thermal treatment. A new structure can then be eventually induced, in contrast to the static patterns arising from the permanent photopolymerization of optical lithography and the irreversible material removal of laser ablation.

To create mesostructures on azopolymers, light-induced mass migration is generally used to morph a smooth surface. The resulting surface pattern is a direct map of the irradiated optical field geometry. Typical experimental configurations involve the use of interfering laser beams and digital holography systems,^17,30−33^ which have been successfully used to fabricate two-dimensional relief patterns, sinusoidal gratings,^30,34^ quasi-crystalline lattices,^35,36^ and even complex planar diffractive optical components.^37−40^

Despite the versatility of the surface patterns that can be created, the structures produced from flat polymer films are limited to less than 2 μm in the vertical direction. This hinders the potential of creating complex 3D features using this illumination scheme. To achieve greater patterning capabilities, alternative approaches involve photoreshaping a pre-existing 3D texture of mesostructures on the azopolymer surface, providing a control that can be extended to several microns in depth.

In most cases, the 3D reshaping approach exploits the sensitivity of azopolymers to the direction of the electric field, where the material migrates parallel to the direction of the light polarization, even in a reversible manner.^41−45^ Structures of high complexity and hierarchical architectures have been successfully reported following this scheme,^37,46^ although many previous studies only emphasize the influence of the light-induced deformation on the two-dimensional section of the reconfigured microvolumes. A first potential example of full 3D structural control has been demonstrated by tailoring the bending radius of micropillars by simply tuning the tilt angle of the incident beam.^10^

Here, we propose a simple framework to control a prestructured azopolymer surface in three dimensions by varying the wavelength of the irradiated light. Although a few studies have paid attention to the potential effects of the light wavelength in the deformation of 3D azopolymer microstructures,^7,10^ we aim to turn this easily tunable optical parameter into a powerful lithographic tool for tailoring the complex 3D structure of azopolymer microvolumes. To this end, we exploit the large variations in absorbance that common azomaterials typically exhibit in the UV–vis region. As a result, different light wavelengths are able to selectively trigger mass migration at different depths within the micropillar volume during the light-induced reshaping process. We focused our study on the analysis of the multiple 3D morphology transitions of an original prestructured azopolymer surface at different wavelengths over increasing light-induced deformations, rationalizing the role of the light penetration depth for the deterministic 3D surface reconfiguration.

To support the potentialities of the 3D structure control for engineering functional surfaces, we demonstrate the tuning of the hydrophobicity of an azopolymer pillar surface by deterministically reshaping the same pristine array into different 3D pillar geometries using light of different wavelengths.

## Results and Discussion

### Penetration Depth as a Lithographic Parameter

The main concept underlying our study is to use the differences in light absorption at different wavelengths as a tool to drive a photoinduced deformation at different depths within an azopolymer microvolume.

**Figure 1:**
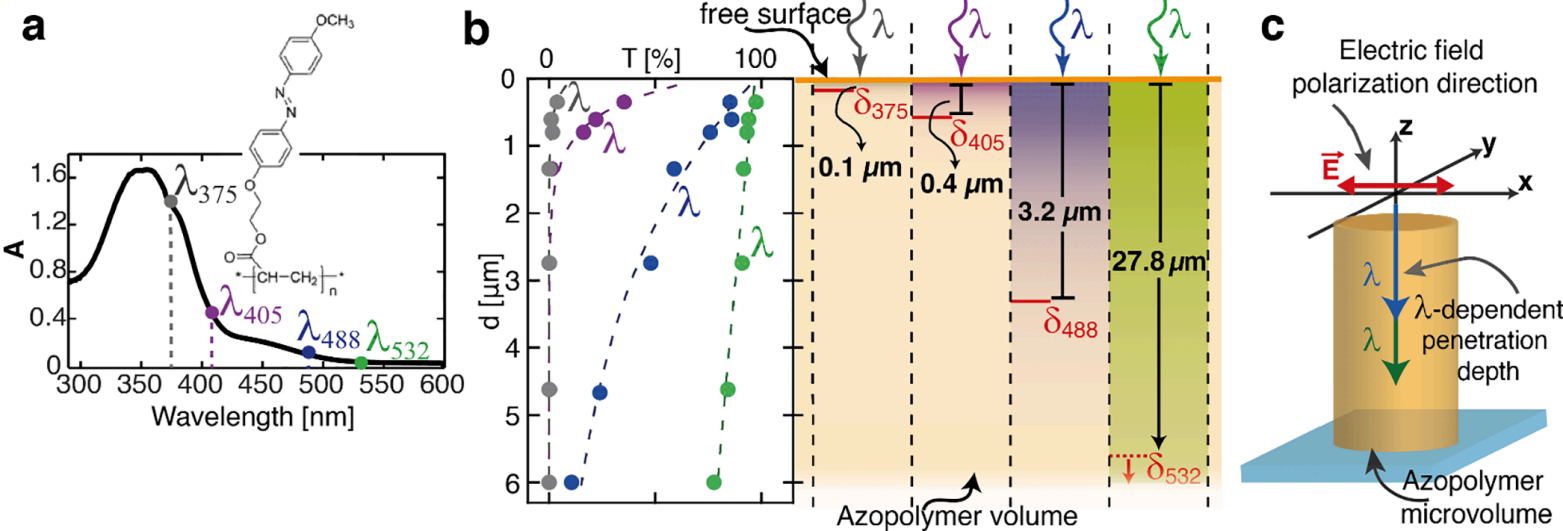
Light penetration depth and 3D reshaping of azopolymer microvolumes. (a) UV–visible absorption spectrum of the azopolymer films used in this study. (b) Left panel: transmittance values measured (dots) at the selected wavelengths for different azopolymer film thicknesses *d*. Dashed lines are the result of data fitting with the LB law. Right panel: illustration of the different penetration depths of light at the selected wavelengths in the azopolymer volume. (c) Scheme of the 3D reshaping of an azopolymer microvolume using different wavelengths of light irradiation. The electric field, oscillating in the *x*-axis, can propagate to different wavelength-dependent positions in the *z* direction, triggering the pillar reconfiguration from different depths in the microvolume.

To this end, we first studied how light of different wavelengths is attenuated as it propagates within the volume of flat azopolymer films of different thicknesses. Figure 1a shows the chemical structure and UV–vis absorption spectrum of a typical film of the azopolymer used in this work. Details about the material synthesis and film preparation are given in the Experimental Section.

The UV/Vis absorption of the azopolymer, which is entirely determined by the optical properties of the embedded azobenzene molecules, exhibits a broad maximum centered around 350 nm and decreases to negligible values above 600 nm, where the polymer becomes essentially transparent. The large variations in absorbance for light from UV (300 nm) to green (550 nm) imply significant differences in the depth that light can propagate within the volume of the film before being completely absorbed, as described by the well-known Lambert–Beer (LB) law.^47^ According to this law, the intensity *I*(*z*) of the light propagating from the surface (at *z* = 0) to the depth *z* within the azopolymer film is attenuated as
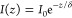
1where *I*_0_ is the intensity at the surface and δ is the light penetration depth. δ defines the location where the incident light intensity is reduced by a factor of 1/e ≈ 0.37. Since it is related to absorption coefficient α(λ) = 1/δ of the dispersive material, the penetration depth depends on the wavelength of the propagating light: δ = δ(λ).

In the present work, we analyzed the effects of different δ(λ) at four discrete wavelengths covering almost the entire absorption region of the azopolymer spectrum (λ_375_ = 375 nm, λ_405_ = 405 nm, λ_488_ = 488 nm, and λ_532_ = 532 nm), as indicated in Figure 1a.

To extrapolate δ_*i*_*≡*δ(λ_*i*_) of our azopolymer at the four λ_*i*_, we measured the UV–vis transmittance spectra *T*(*d*) = *I*(*d*)/*I*_0_ of flat films with different thicknesses *d* between 0.3 and 6.0 μm (see Experimental Section). The results are presented for each λ_*i*_ as colored dots in the plot in the left panel of Figure 1b. The corresponding penetration depths δ_*i*_ were obtained by fitting the data with respect to the film thickness with the LB law in eq 1 (dashed lines in Figure 1b). The values of δ_*i*_ are presented in Figure 1b (right panel), together with a quantitative graphical visualization of the light attenuation in the azopolymer volume for a direct comparison of the different behaviors in the exponential intensity decay with the wavelength. As expected, the analysis showed significant variations of the penetration depths, differing up to more than 2 orders of magnitude at the boundaries of the tested absorption region (δ_375_ = 0.1 μm; δ_532_ = 27.8 μm).

Since the layers of an azopolymer volume reached by non-negligible light intensity can be subjected to a photodeformation, as the next step of our analysis, we analyzed how the varying penetration depth can affect the geometry of an azo microvolume. In general, according to previous studies,^17,46^ the microvolume photoreconfiguration process requires two conditions to work effectively. The first condition is an irradiation configuration with a polarized light field that has a vectorial component in the plane of one of the microvolume surfaces. The second is the presence of a sharp boundary, identifying a volumetric discontinuity, in the surface under consideration to observe a global deformation along the direction of light polarization.^46^ Maximum deformation efficiency is achieved when the polarization direction is orthogonal to the boundaries.^48,49^

These two conditions are typically met in the experiments by irradiating an array of cylindrical or square micropillars with linear polarized light at normal incidence to deform the top surface (in the *x–y* plane) of the microvolume, as shown schematically in Figure 1c. However, with the ability to reach different depths within a microvolume, light of different wavelengths can also deform the lateral surfaces of the microvolume differently, even in this simple illumination configuration. In fact, the electric field of the light propagating a considerable distance *z* within the volume, as happens for wavelengths with large penetration depths, has a nonzero component orthogonal to the volume boundary (the lateral surface of the micropillar). This can then trigger a material displacement in the *x–y* plane from different depths.

In this scenario, the effect on the final 3D geometry depends directly on the penetration depth and then on the light wavelength, in addition to other conventional parameters such as the polarization state and angle of incidence of the irradiated beam.

**Figure 2:**
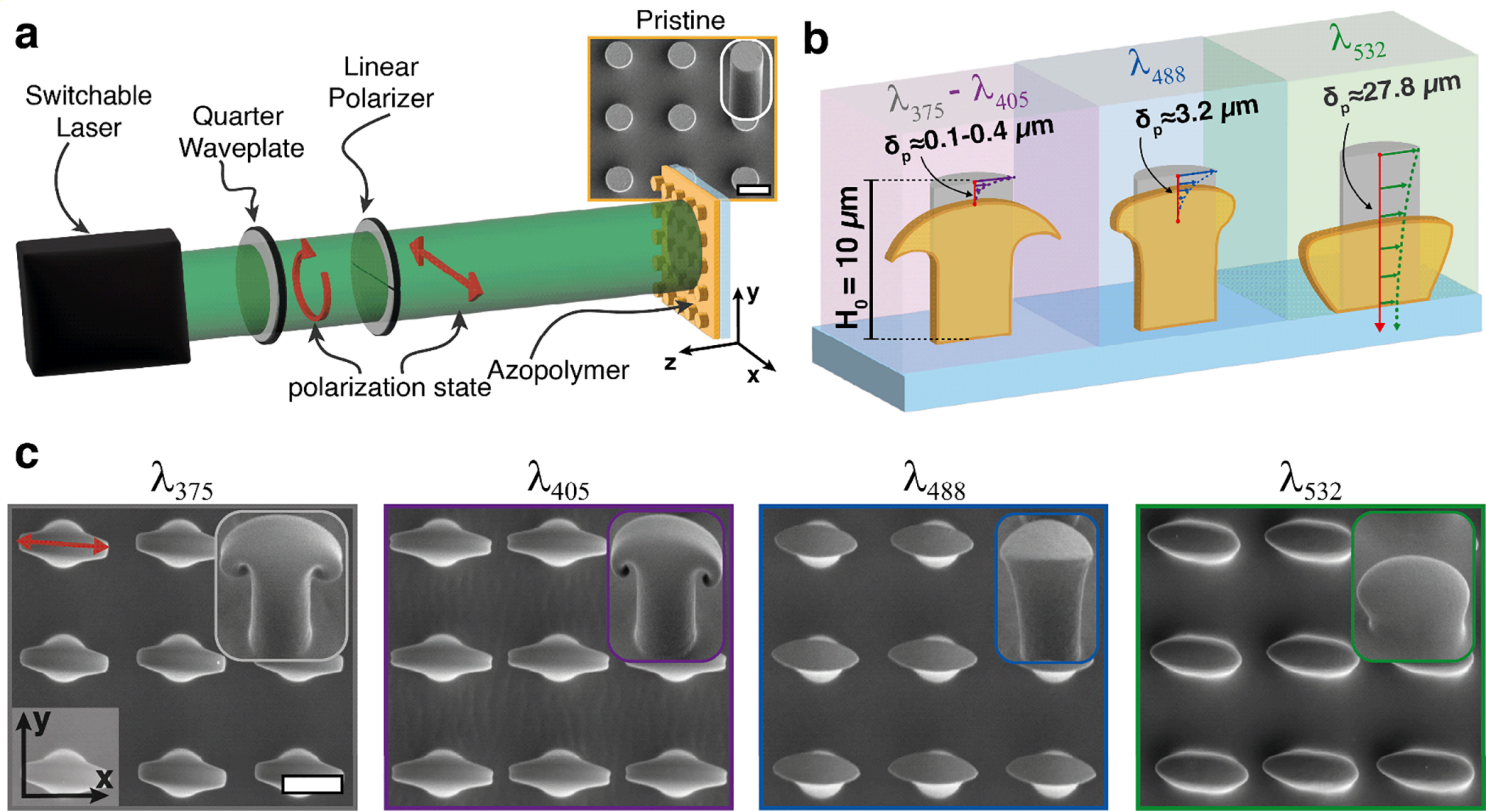
Irradiation setup and the morphology of the reshaped micropillars at selected wavelengths. (a) Irradiation scheme of the experiment. The top- and side-view SEM images in the inset show the morphology of the pristine surfaces used in the experiment. (b) Qualitative illustration of the decaying intensity profiles of the selected wavelengths and their effect on the 3D geometry of the reconfigured pillars. (c) SEM images of the top view of the reshaped micropillars at the selected wavelengths. The red arrow in the first panel indicates the direction of light polarization. The insets show a side view of a corresponding micropillar. The scale bar is 5 μm.

To quantitatively characterize the effect of the light wavelength on the 3D shape of the azobenzene microvolumes, we designed an experiment with the same configuration as described in Figure 1c. Four different and copropagating diode lasers at the wavelengths λ_*i*_ were used to irradiate a prepatterned azopolymer surface. The collimated beams from each laser passed through a broadband quarter-wave plate and a fixed linear polarizer, before impinging orthogonally on the sample plane with a linear polarization in the *x-*direction. The optical setup is shown in Figure 2a, where the different lasers used in the experiment are schematized as a single switchable laser for simplicity.

The prepatterned azopolymer surfaces were prepared as a square array of cylindrical micropillars, fabricated by standard soft lithography (see Experimental Section for details). A micropillar template with a height *H*_0_ = 10.0 ± 0.2 μm was chosen to investigate different scenarios where the penetration depth was much shorter, comparable, or longer than the initial height of the microvolumes. The pillar diameter and the array periodicity were 5.0 ± 0.2 and 10.0 ± 0.2 μm, respectively.

The light-induced reconfiguration experiments were performed by exposing identical films with the prepatterned micropillar surface to each wavelength λ_*i*_. The polarization and the angle of incidence were kept fixed throughout the experiment, while the exposure dose was increased to obtain tunable degrees of deformation in the direction of the light polarization^50^ (see Experimental Section for details).

As a first analysis, we characterized the morphology of the photoreconfigured surfaces to compare the different 3D geometric evolution of the micropillars irradiated with the different wavelengths with the empirical mechanism illustrated in Figure 2b. Based on the ratio of the pristine height *H*_0_ of the azo micropillar to the measured penetration depths δ_*i*_, we expected at least three different families for the geometry of the deformed microstructures, as schematized in Figure 2b, whose 3D morphology is determined by the different fractions of the original volume able to move effectively under different wavelength irradiation. We used scanning electron microscopy (SEM) to characterize the surface morphology of the samples after the exposures (see Experimental Section). Figure 2c shows the top-view SEM images of the reconfigured micropillars. As anticipated, light irradiation produced asymmetric structures that appeared elongated in the direction of the polarization. Longer exposure times produced larger surface elongation (see also Figure S1), but the apparent in-plane morphology is similar regardless of the wavelengths used.

This behavior has been exploited in several studies that aimed at producing light-induced anisotropic microstructures on the azopolymer surface,^50^ where the actual 3D pillar morphology was not of primary interest. However, the SEM side views of the micropillars in the insets of Figure 2c show significantly different 3D geometries, in agreement with the hypothesis in Figure 2b. More specifically, λ_375_ and λ_405_ produced similar 3D micropillar shapes with strong overhanging features. For these two sets of microstructures, the deformation was confined to a region close to the top surface of the micropillars, resulting in a curled shape on both sides of the micropillars appearing already after a small exposure dose. Very different results were obtained for the structures irradiated at λ_532_. In this case, the photodeformation involved the entire micropillar volume, resulting in shorter and larger micropillars elongated in the *x-*direction even at the bottom surface. Finally, light at λ_488_ caused a relatively intermediate 3D deformation regime that involved the displacement of a significant fraction of the initial microvolume but preserved a clear, pristine cylindrical structure at the bottom surface.

### Morphological Analysis of 3D Reshaped Azopolymer Micropillars

**Figure 3:**
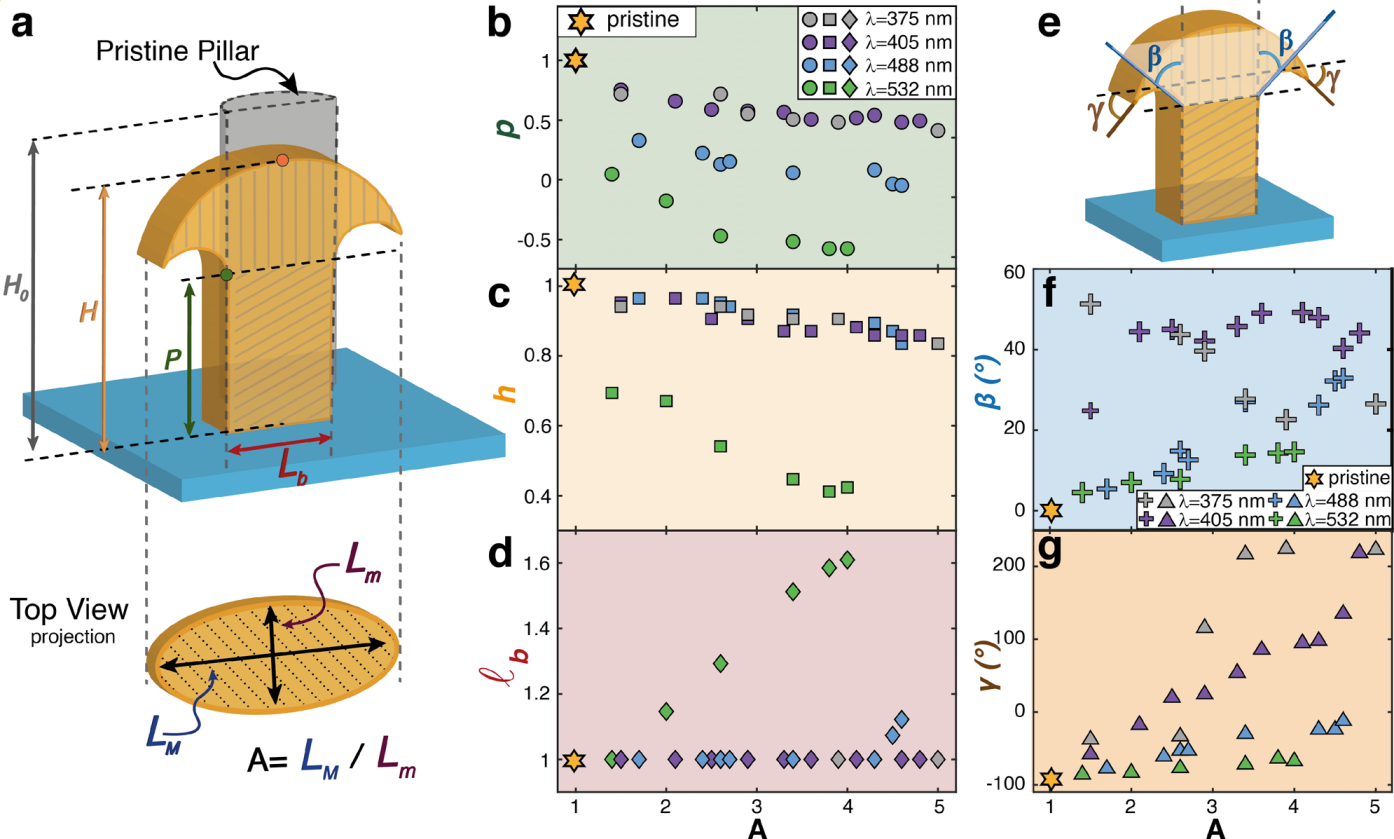
Wavelength-dependent evolution of the geometric pillar parameters. (a) Schematics of the 3D structure for the definition of the deformation morphological parameters. (b–d) Evolution of the parameters *p*, *h*, and *l*_b_ as a function of *A*. (e) Definition of the angle-based deformation parameters. (f, g) Evolution of the aperture angle β and the curling angle γ as a function of strain *A*.

To quantitatively describe the evolution of the 3D geometry of the micropillars irradiated with the different wavelengths and increasing degree of deformation, we conceptualized a set of measurable geometric parameters (*p*, *h*, *l*_b_, β, γ) capable of capturing the essential features of the differently photoreconfigured microstructures, as schematically shown in Figure 3a,e. Each of these parameters can be measured from the SEM images, as shown in Figure S2, and their geometrical meanings will be discussed individually below.

In the experiments, only the exposure dose was tuned to achieve a gradual increase in microvolume deformation. Due to the large differences in the total light absorption of the azopolymer, the deformation dynamics were very different for the different wavelengths. For a reliable comparative analysis, all geometric parameters were then analyzed as a function of the *x*–*y* strain (*A*) of the top surface, which we were able to tune in the same range for all the wavelengths. The strain *A* was calculated from the top-view SEM images as the ratio of the major axis *L*_M_ to the minor axis *L*_m_ of the approximately elliptical top section of the deformed micropillars (Figure 3a). This choice allows one to directly attribute the differences in the 3D morphology of the photodeformed structures with similar in-plane anisotropy to the light penetration depth of each wavelength, independent of the temporal dynamics required to induce the deformation.

#### Pristine Residual Fraction: Parameter *p*

The parameter *p*, defined in Figure 3a, characterizes the fraction of the volume of the original cylindrical micropillar that remains undeformed during the photoreconfiguration process. It is measured as the ratio of the distance from the pillar base in the vertical direction where the light-induced deformation is initiated (*P*) to the pristine pillar height, *H*_0_ (*p* = *P*/*H*_0_).

Different light penetration depths directly affect the parameter *p*. A value of *p* close to 1 indicates that only a small portion of the original volume is deformed, while a value close to 0 implies that the entire pristine structure is reconfigured. Experimentally, the distance *P* was measured from side-view SEM images by identifying the intersection point (green dot in Figures 1a and S2a) of a line drawn tangent to the lateral pillar surface in the deformed region and the vertical direction. Thus, *p* can take negative values, meaning that the intersection point occurs at a position below the base of the pristine micropillar.

Figure 3b shows the evolution of the parameter *p* measured as a function of strain *A* for the four wavelengths λ_*i*_. Although the dynamics are different, the analysis shows a decreasing trend for *p* with increasing *A* at all of the wavelengths. This observation is a direct consequence of the different light penetration depths and the approximate volume conservation in the light-induced reconfiguration process of azopolymers.^8^ In fact, as the material layers close to the top surface start to migrate laterally along the electric field direction, the underlying layers in the micropillar volume can be reached by the previously absorbed light and then move in turn. This allows the light to penetrate deeper into the micropillars, further deforming the deeper layers, which are characterized by a smaller *p.*

More specifically, the evolution of the parameter *p* at different λ_*i*_ supports the 3D morphological analysis in Figure 2b,c. The reconfiguration of the pillars at λ_375_ and λ_405_, both characterized by a light penetration depth that is less than 5% of the initial height of the azo microvolume, resulted in similar final 3D geometries and similar residual undeformed volumes, as confirmed by the overlapping trends for *p* in Figure 3b. Illumination at λ_488_ resulted in a faster decay of *p*, which involves half of the initial volume already for *A* < 2.0, in agreement with the larger light penetration depth. In contrast to the others, the parameter *p* for the pillars illuminated at λ_532_ decreases from the value *p* ≈ 0 and becomes negative for *A* ≈ 2.0. This suggests that the entire volume of the micropillars was immediately reconfigured according to the large light penetration depth. The value of *p* ≈ 0 is also reached by the pillars illuminated at λ_488_ after a strong reconfiguration process (*A* ≈ 4.5).

#### Reduction of Pillar Height and Base Deformation: Parameters *h* and *l*_b_

The parameters *h* and *l*_b_ represent the percentage of height reduction *h* = *H*/*H*_0_ and the basis deformation *l*_b_ = *L*/*l*_0_ of the photodeformed pillars with respect to the pristine microcylinders (Figure 3a). The analysis of the trends of these two structural parameters with the deformation strain *A* strengthens the understanding provided by parameter *p* for characterizing the evolution of the 3D geometry with the wavelength. As shown in Figure 3c, the parameter *h* of all wavelengths gradually decreases as the elongation of the top surface of the pillars becomes more pronounced. This further confirms the volume conservation mechanism of Figure 2b, where a fraction of the volume is gradually transported to the side in the reconfiguration process, thus, reducing the actual height of the micropillars. The strong morphological difference induced by the irradiation at λ_532_ in Figure 2b is also emphasized by the rapid decrease of its *h* trend compared with the others, caused by a much larger volume of material involved in the deformation. In addition, at λ_532_ the base of the pillars was also deformed due to the large penetration depth, as described by the increase in the parameter *l*_b_ reported in Figure 3c. This is consistent with the negative values of the *p* parameter measured at λ_532_ for *A* > 2.0 (Figure 3b). As expected from the small light penetration depths (compared to *H*_0_), wavelengths λ_375_ and λ_405_ did not reach the pillar base at any deformation strain, resulting in a constant *l*_b_ ≈ 1.0. Finally, the intermediate penetration depth for the illumination at λ_488_ affected the pillar base only for large volume deformations (*l*_b_ increased only for *A* > 4.0), consistent with the concurrent observation of *p* ≈ 0.

#### Aperture Angle and Surface Curling: Parameters β and γ

The previous analysis shows similar deformation parameter trends for micropillars reconfigured with λ_488_ (δ_488_ ≈ 3.2 μm) and λ_375_/λ_405_ (δ_375_ ≈ 0.1 μm and δ_405_ ≈ 0.4 μm), although the considerable difference in the light penetration depth and the resulting 3D morphology is shown in Figure 2c.

This is because parameters *p*, *h*, and *l*_b_ are not sufficient to capture the difference in the curvature of the deformed portions of the microstructures, which requires the introduction of two angle-based parameters (β and γ), as defined in Figure 3e.

The β parameter represents the aperture angle of the primary deflection of the lateral pillar surface with respect to the vertical axis. This is the angle of an ideal trapezoid that could roughly approximate the actively deformed volume of the micropillars above point *P* (Figures 3e and S2). The pristine pillars have an aperture angle of β = 0°, while the deformed pillars are characterized by increasing values of β.

The parameter γ measures the deviation of the shape of the structure edges from the ideal trapezoid, characterizing then the deflection of the curling edge (Figure 3e) with respect to the *x–y* plane (for which γ = 0°). In our sign convention, the upward curling is characterized by γ **<** 0° and downward curling by γ **>** 0°. The pristine cylindrical pillars have γ **= –** 90°, while γ = **–** β for an ideal trapezoidal shape.

The evolution of parameters β and γ with *A* for all wavelengths is shown in Figure 3f,g.

As can be seen from the plot in Figure 3f, the angle β measured for the deformed structures at λ_532_ and λ_488_ shows relatively monotonically increasing trends, with the higher slope for λ_488_ arising from the higher vertical intensity gradient in the pillar volume due to the shorter penetration depth. The angle β for λ_405_ and λ_375_ follows an irregular trend due to the curling of the micropillar top surface, which makes the trapezoidal approximation still insufficient. However, the abrupt increase of β in the early evolution stages (*A* < 2.0) shows that a wavelength with a much shorter penetration depth than the *H*_0_ provides a rapid and intense curvature of the edges of the top surface of the microvolume.

The evolution of the parameter γ can be divided into two categories, i.e., the *slow-curling* structures obtained at λ_532_ and λ_488_, and the *fast-curling* structures obtained at λ_405_ and λ_375_.

As shown in Figure 3e, the slow-curling structures are characterized by γ **<** 0° for each value of *A*. On the other hand, the fast-curling structures undergo a transition to γ **>** 0° already at small strain values, producing the characteristic 3D geometry presented in Figure 2c. The curling of the surface can be empirically ascribed to the different speeds at which successive thin layers in the azopolymer microvolume can potentially move in the lateral direction due to attenuation of the light intensity. In particular, the layers close to the top surfaces move faster than the underlying layers with a difference that increases with shorter penetration depths. The interfacial forces between the different layers within the azopolymer volume can then cause the surface to curl if the speed variation is large enough. This situation is verified in the case of the fast-curling structures at λ_405_ and λ_375_, differently from the structures deformed at λ_532_ and λ_488_, which instead result in a negligible curling at any deformation level.

#### Control of the 3D Pillar Morphology

The accurate morphological analysis of the previous sections can provide the ability to deterministically fabricate different complex 3D mesostructures by reconfiguring the azopolymer micropillar array with different illumination configurations and light wavelengths. It must be clarified that the evolution of the 3D geometric parameters is entangled, mainly due to the volume conservation that occurs in the light-induced azopolymer reconfiguration process. However, according to our results, the accurate selection of the light wavelength can provide a strategy to minimize the parameter entanglement, thus achieving straightforward control over various complex 3D mesostructure shapes. In addition, it is important to note that the technique presented here is not limited to the simple experimental scheme presented here. Other irradiation states, such as different polarization configurations, the inclusion of additional spatial constraints on light-induced mass migration, like a polydimethylsiloxane (PDMS) cap layer,^17,46^ and different morphologies of azo microvolumes can be explored to generate a variety of different 3D structures.

### Wavelength-Tunable 3D Surface Photoreconfiguration for Hydrophobicity Tailoring

To highlight the suitability of our results in surface engineering, we used the 3D light-induced reconfiguration of a single pristine array of micropillars at different wavelengths to tailor the surface hydrophobicity.

The study of the influence of the 3D mesoscopic roughness to control the wetting properties of solid surfaces is an area of intense research. Along with the conventional models (Wenzel and Cassie–Baxter) that relate the wetting behavior to the geometric parameters (e.g., pitch, diameter, height) of the surface textures of a given material, many studies have highlighted the influence of sharp overhanging and re-entrant features that are capable of pinning the triple-phase contact line^8,51^ and inducing superhydrophobic effects.^52,53^

For example, the reduction in the height of the pillars sustaining the roughness-induced hydrophobic wetting Wenzel state can cause a decrease in the hydrophobicity observed as a smaller contact angle (CA).^54,55^ The induction of overhanging structures, on the other hand, can favor the trapping of air between the solid structures in a Cassie–Baxter state, inducing an increase in hydrophobicity and CA. Producing similar morphological variations on artificial rough surfaces typically requires dedicated advanced manufacturing methods that cannot be adapted to achieve opposite wetting behaviors (e.g., both increase and decrease hydrophobicity).

With the demonstrated ability to generate different 3D shapes starting from a single pristine microvolume geometry, the light-induced reconfiguration of azopolymer mesostructures may represent a versatile approach to optically tune the surface wettability in untrivial situations. To this end, we used the reconfiguration of an array of cylindrical azopolymer micropillars at two wavelengths to selectively induce an increase or decrease in the CA of the pristine surface.

For the experiment, surfaces prepared with similar geometry as in Figure 2 but different arrangement (diameter = 8.0 ± 0.2 μm, pitch = 12.0 ± 0.2 μm, *H*_0_ = 3.0 ± 0.2 μm) were irradiated with circularly polarized collimated laser beams at λ_375_ and λ_488_ to induce isotropic in-plane pillar reconfiguration.^46^ The intensity of the lasers was adjusted to achieve similar reconfiguration dynamics at the two wavelengths (see Experimental Section). We used different exposure times to produce increasing lateral deformation, while the different penetration depths of the irradiation differently deformed the volume of the pillars. It should be noted that the penetration depth of λ_488_ was comparable to the *H*_0_ of the new pristine array, while the penetration depth of λ_375_ was still much shorter. To enhance the formation of a sharp and thin re-entrant instead of curled features, the top surface with irradiation at λ_375_, we covered the pristine pillar array with a thin transparent layer of flat PDMS during exposure. To characterize the wetting properties of the pristine and the photoreconfigured surfaces, we measured the CA of 2 μL water droplets with a custom optical system, while SEM analysis was used to characterize the morphology of the reconfigured surfaces (see Experimental Section).

**Figure 4:**
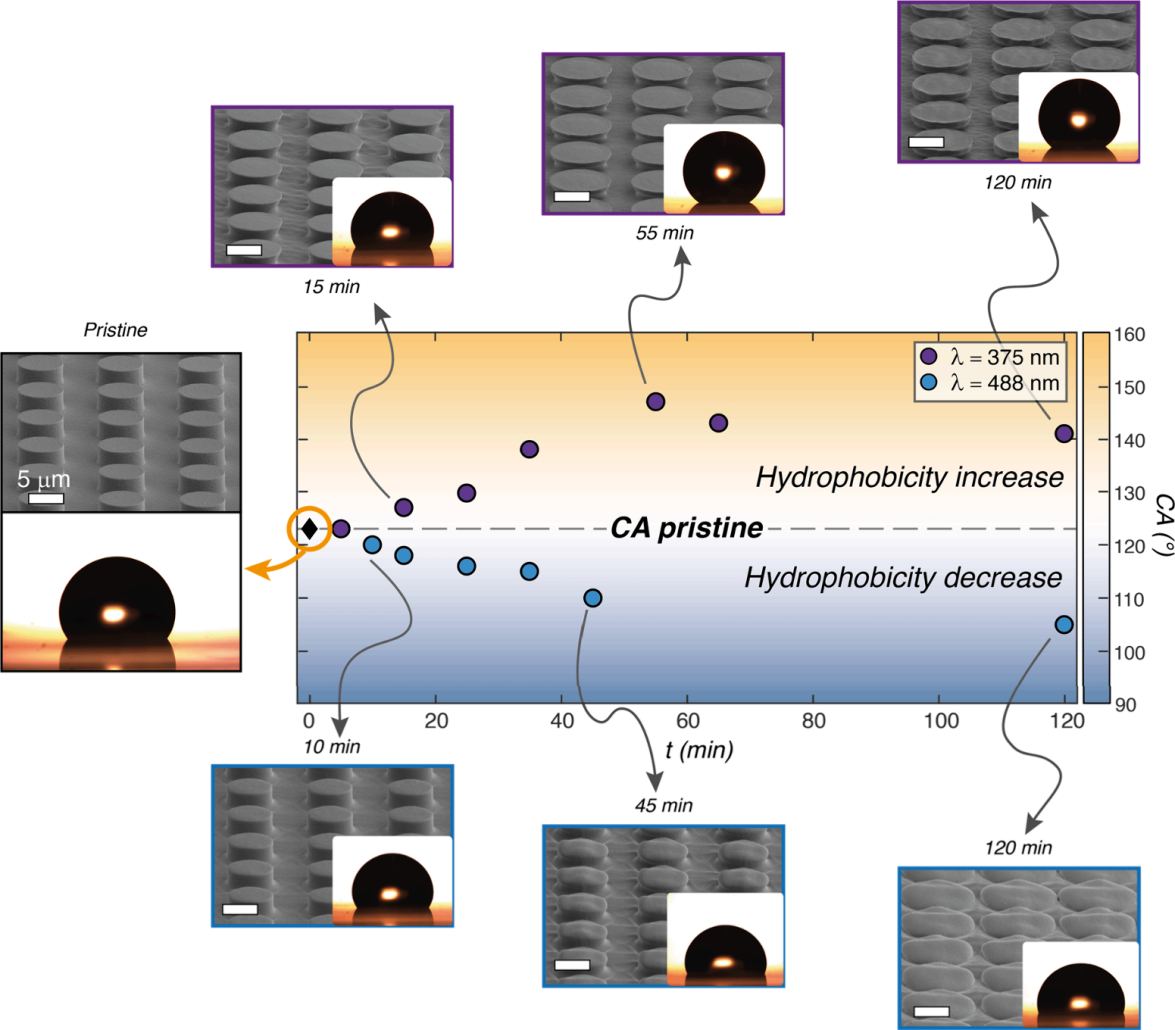
Tuning of hydrophobic behavior. The graph shows the evolution of the contact angle (CA) of the micropillars irradiated at λ_375_ (top) and λ_488_ (bottom) with increasing irradiation time (*t*). For the pristine surface and selected CA values, the tilted SEM images of the 3D reshaped micropillars and their corresponding water CA photographs are shown as a representation of the different regimes. All white scale bars are 5 μm.

Figure 4 shows the evolution of the surface morphology and the relative measured CA for increasing exposure times at the two selected wavelengths. Two distinct wetting behaviors are clearly identified for the two sets of surface morphologies produced by the different irradiated wavelengths. The reconfiguration at λ_375_ produced mushroom-like pillars with increasing diameter. According to the analysis of the residual undeformed volume fraction in the previous section, the short penetration depth of the light allowed the preservation of the pillar-like structure and the formation of sharp re-entrant features. The measured CA increased from the value of CA_p_ = 123 ± 3° of the pristine array, whose wetting state is dominated by a pinned state dependent on the geometrical parameters of the pillars,^8^ to CA = 147 ± 3° after 55 min of exposure, according to the expected enhancement of the Cassie–Baxter wetting state. The decrease in CA from the maximum value observed for longer exposure times is due to the decrease in pillar height (as in Figure 3c), which could favor the filling of the air gaps between the pillars with the liquid.

An opposite trend for the CA was observed for the surfaces reconfigured at λ_488_, where the absence of re-entrant features and the significant height reduction, produced by the greater light penetration depth, favored the transition from the pinned pristine state toward the Wenzel hydrophobic state, until reaching CA = 105 ± 3° after 120 min of exposure. At this stage, a complete deformation of the micropillars was observed, transforming the pristine cylindrical geometry into flattened dome-like structures. This behavior is consistent with the expected microvolume reconfiguration produced by a light beam with penetration depth comparable to the pristine pillar height. As a result of the analysis in Figure 4, the CA on the considered micropillar azopolymer surface can be precisely tuned in a range of more than 40° by exploiting the light penetration depth as a lithographic parameter.

## Conclusions

In this study, we have demonstrated the wavelength of the light, which translates to the penetration depth, as a new lithographic parameter for additional control in the 3D photoinduced structuring of azopolymer microvolumes. By quantitatively analyzing the 3D geometry of a single pristine micropillar array reconfigured with laser beams of different wavelengths, we have shown a predictable control on different photodeformation evolutions. The final microvolume geometry can also be tuned by simply changing the illumination configurations, such as the polarization state and the exposure dose. Completely different 3D architectures with similar isotropic and anisotropic in-plane deformations have been obtained, with the irradiated light penetration depth being the main discriminating parameter.

To support the wide potential application of this wavelength-dependent 3D microvolume reconfiguration in the fabrication of functional surfaces, we successfully demonstrated the tuning of the hydrophobicity of an original pristine array in a large range of water CA values by simply deforming the microstructures with two different light wavelengths.

Compared to other techniques, the 3D-shaping of azo-microvolumes relies on moving a fraction of the azopolymer volume to a new configuration in a straightforward manner, which can produce multiple new 3D architectures without adding or removing material from the original structure. This process avoids the use of any multiple steps, post-treatment, and additional materials in the process to achieve geometric variations demanded by the specific application. In addition, the reconfiguration process does not require a high-energy laser. A low-cost generic monochromatic illumination source, such as commercially available LED lamps coupled with narrow-band filters, can produce the same result as that in this study. Our analysis paves the way for the realization of a cost-effective and scalable method to fabricate functional 3D mesostructures with engineered optical, mechanical, and structural properties based on the azopolymer photoreconfiguration.

## Experimental Section

### Azopolymer Synthesis

The azopolymer was synthesized by radical polymerization of the photoresponsive monomer (E)-2-(4-((4-methoxyphenyl)diazenyl)phenoxy)ethyl acrylate, according to previously reported procedure.^29^ The chemical structure of the polymer is schematized in Figure 1a. Reagents for the synthesis were purchased from Merck and used without further purification. The UV–visible absorption spectrum shown in Figure 1a was recorded by using a Jasco V560 spectrophotometer. Further details of the azopolymer synthesis, thermal analysis, and molecular weight distribution were reported in previous studies.^8^

### Penetration Depth Measurement

UV–visible transmittance spectra, which were used to extrapolate the penetration depth δ_*i*_ = δ(λ_*i*_) at the different wavelengths (Figure 1b), were recorded with a PerkinElmer Lambda 900 spectrophotometer on transparent amorphous thin films with thicknesses varying from 0.3 to 6 μm. The films were prepared by spin-coating the polymer solutions in 1,1,2,2–91 tetrachloroethane onto glass slides by accordingly varying the polymer solution concentration and rotation speed from 80 mg/mL and 2500 rpm to 180 mg/mL and 300 rpm.

### Prestructured Surfaces Fabrication

Azo-micropillar arrays were fabricated by using a replica molding process that involved two sequential steps: fabrication of the mold containing the negative pattern and the transfer of the pattern to the azopolymer surface.

#### PDMS Mold Preparation

A silicon wafer with a cylindrical micropillar array arranged in a square lattice (height *h* = 10.0 ± 0.1 μm, diameter *d* = 5.0 ± 0.1 μm, pitch *p* = 10.0 ± 0.1 μm) was used as master template for the molding process. Prior to use, the silicon wafer underwent an antistick treatment by exposing its surface to silanizing vapors (trichloro(1 H,1 H,2 H,2 H-perfluorooctyl)) for 90 min at 125 °C in an airtight glass container. The container was then left open for another 90 min at 150 °C to ensure the removal of vapor residues. The PDMS used for mold fabrication was prepared by blending the elastomer (Sylgard 184, Dow Corning) with a curing agent in a 10:1 weight ratio and placing it in a vacuum chamber to remove the air trapped in the mixture during the blending process. The obtained mixture was then gently poured onto the silicon wafer and cured at 80 °C for 2 h. Finally, the solidified PDMS was carefully detached from the wafer, obtaining the negative pattern of the micropillar array.

#### Azo-Micropillar Array Fabrication

A few drops of a 10 wt % solution of the azopolymer in 1,1,2,2–91 tetrachloroethane were placed on a clean coverslip, and the PDMS mold was gently placed on top of the solution drops, allowing the solution to fully spread into the negative pattern of the micropillar array. The system was left at room temperature for 5 h to allow the solvent to completely evaporate through the PDMS micropores. Finally, the PDMS mold was carefully detached, producing the desired micropillar array pattern on the surface of the azopolymer film surface.

### Optical 3D-Shaping of Micropillars

Four chromatic light sources were used to selectively illuminate the pristine micropillars: 375 nm (Oxxius LBX 375), 405 nm (Coherent OBIS 405 LX), 488 nm (Coherent OBIS 488 LS), and 532 nm (Laser Quantum Opus 532). The intensities of the lasers were tuned to compensate for the large difference in the deformation dynamics due to the total light absorption of the azopolymer at different wavelengths. The intensities measured at the sample plane are as follows: *I*_375_ = 90 mW/cm^2^; *I*_405_ = 70 mW/cm^2^; *I*_488_ = 160 mW/cm^2^; and *I*_532_ = 4 W/cm^2^. Each irradiation experiment was performed with collimated beams of approximately a 4.0 mm diameter. The exposure times were chosen in the range from 2 to 22 min in order to obtain comparable *x*–*y* strain (*A*) of the top surface. See Figure S1 for the complete structuring evolution.

#### Optical Setup

Figure 2a shows the schematic of the optical setup: after selecting the appropriate light source, the beam was linearly polarized by a quarter-wave plate and a linear polarizer. Finally, the beam was incident normally on the sample surfaces (placed on the **x*–*y** plane).

### Characterization of the 3D Micropillar Structure

#### Morphological Characterization

A scanning electron microscope (SEM), FEI Nova NanoSEM 450, was employed to investigate the morphology of the micropillars. A nanometric layer of a Au/Pd alloy was sputtered on the sample surface using a Denton Vacuum Desk V TSC coating system prior to the SEM analysis. The top-view images were collected using a magnification 5000×. The side-view images were acquired at magnification 9000× by tilting the sample by 45° around the *x-*axis.

#### Evaluation of the Structural Parameters

Graphical analysis to measure the geometric parameters from the SEM images (Figure S2) was performed using the *ImageJ v1.46r* software. An average of at least 20 micropillars were used to estimate each structural parameter.

### Hydrophobicity Tailoring

A cylindrical micropillar array geometry with *h*= 3.0 ± 0.2 μm, *d* = 8.0 ± 0.2 μm, and *p* = 12.0 ± 0.2 μm was chosen for the hydrophobicity tailoring study. The surfaces, prepared as described in the previous section, were three-dimensionally reshaped using light at two selected wavelengths: 375 and 488 nm. The water CA was measured for the pristine and reshaped surfaces.

#### 3D-Shaping of Micropillars for the CA Experiment

A circularly polarized light configuration was used for both light wavelengths. A telescopic configuration was used to obtain a suitable structured area to place the water droplet for accurate evaluation of its CA. During the exposure to the light at λ = 375 nm, the pristine surface was primarily covered with a PDMS film on top of the micropillar array. The micropillars were reshaped for different exposure times in the range of 5–120 min in order to evaluate the evolution of the wettability properties. The light intensity was 50 mW/cm^–2^ for both wavelengths.

#### CA Experiment

A homemade experimental setup was used for the water CA measurements. A 2 μL of water droplet, provided by a Hamilton microliter syringe, was carefully placed on the sample surface and imaged with a CCD after approximately 5 s in a temperature–humidity controlled room. The CA (left and right) of each drop was measured using *ImageJ v1.46r* software with the *DropSnake plug-in*. An average CA value was extracted from at least five independent measurements for each structured area.

## Supporting Information Available

SEM images and plot of the evolution of the 2D deformation with the exposure time; graphical parameter definitions for morphological analysis (PDF)
