# Supplementary material for: Wavelength-Dependent Shaping of Azopolymer Micropillars for Three-Dimensional Structure Control
Source: ACS Appl Mater Interfaces. 2023 Aug 30;15(36):43183–92. doi: 10.1021/acsami.3c09264 (PMC10510105; doi:10.1021/acsami.3c09264)
Supplement: Supplementary file 1 — am3c09264_si_001.pdf [file am3c09264_si_001.pdf]

Supporting Information:

Wavelength-dependent shaping of azopolymer micropillars for three-dimensional structure control

I Komang Januariyasa[†], Fabio Borbone[‡], Marcella Salvatore [,†,§], Stefano L. Oscurato[†,§]*

[†] Department of Physics "Ettore Pancini", Università degli Studi di Napoli "Federico II", Via Cintia 21, 80126, Naples, Italy

[‡] Department of Chemical Sciences, Università degli Studi di Napoli "Federico II", Via Cintia 21, 80126, Naples, Italy

[§] Centro Servizi Metrologici e tecnologici Avanzati (CeSMA), Università degli Studi di Napoli "Federico II", Corso Nicolangelo Protopisani, 80146, Naples, Italy

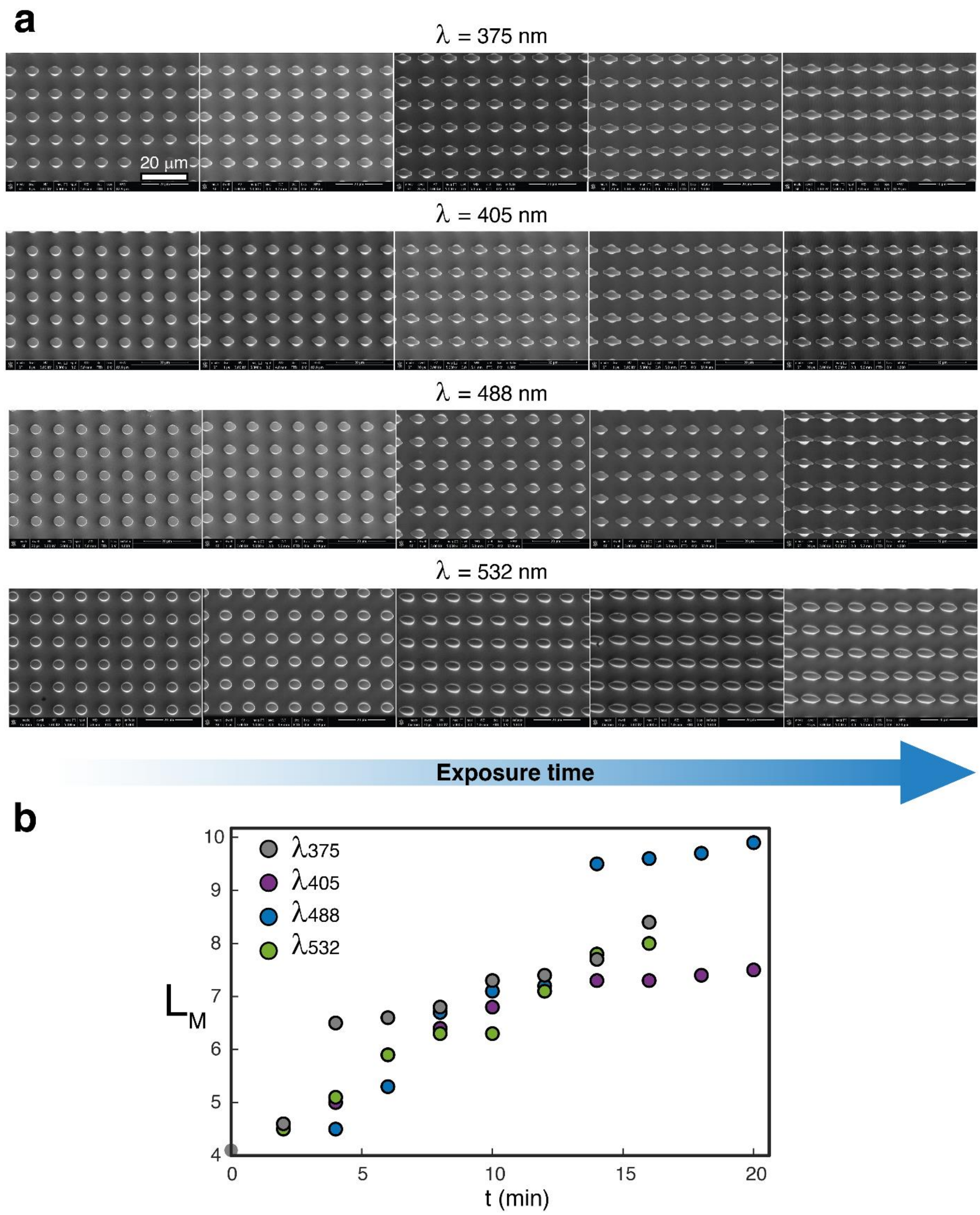

Figure S1. The evolution of the 2D deformation with the exposure time. (a) SEM images of the top view of the micropillars deformed with each selected wavelength increasing the exposure time. (b) The temporal evolution of the major axis of the approximately elliptical top section of the deformed micropillars (L_M) for the selected wavelengths.

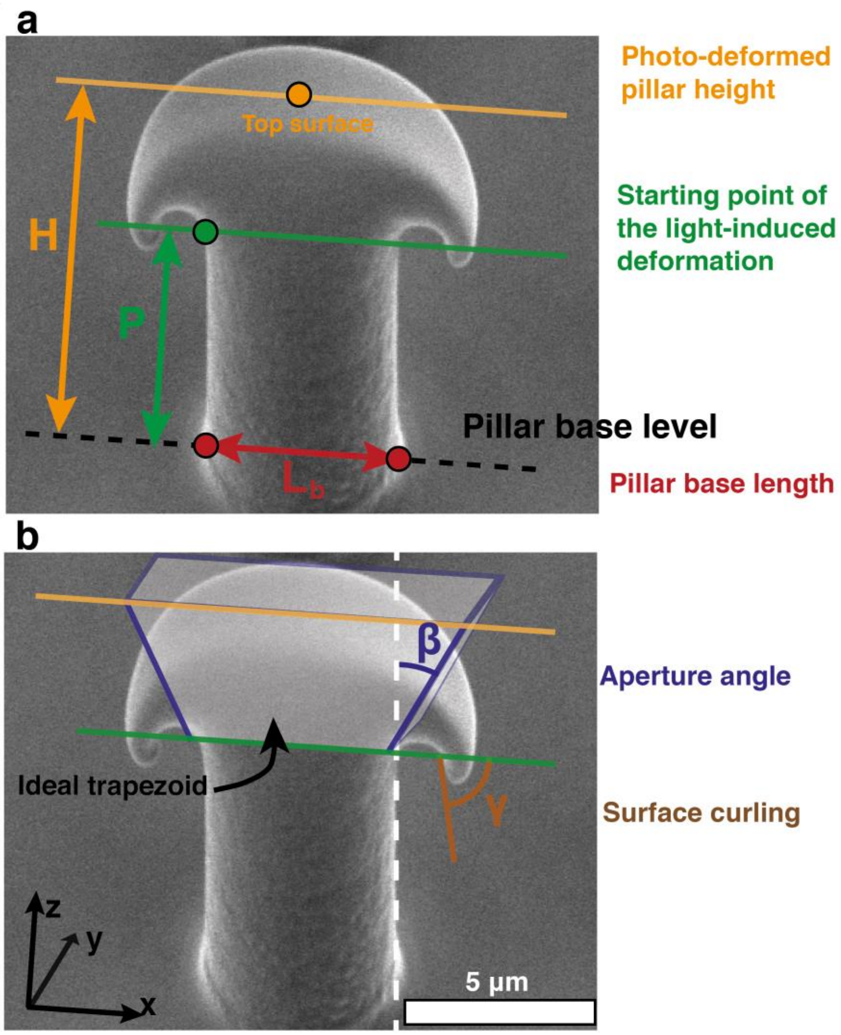

Figure S2. Parameter definitions for morphological analysis. SEM images of a micropillar exposed for 16 min at λ=405 nm. (a) Measurement path of length-based parameters: *P* - distance from the pillar base in the vertical direction to the starting point of light-induced deformation; *H* – distance from the pillar base in the vertical direction to the top surface; L_b – pillar base length corresponding to the undeformed micropillar, in the image shown. (b) Measurement path of angle-based parameters. An ideal trapezoid, that approximate the actively deformed volume of the micropillars above the point *P,* was used to define: β – the aperture angle of the primary deflection of the lateral pillar surface with respect to the vertical axis; γ – the deviations of the shape of the structure edges from the ideal trapezoid, characterizing the deflection of the part of the deformation with respect to the x-y plane.
